# Fabrication of PLA–Date Fiber Biocomposite via Extrusion Filament Maker for 3D Printing and Its Characterization for Eco-Friendly and Sustainable Applications

**DOI:** 10.3390/polym17192707

**Published:** 2025-10-08

**Authors:** Syed Hammad Mian, Abdulrahman bin Jumah, Mustafa Saleh, Jabair Ali Mohammed

**Affiliations:** 1 Advanced Manufacturing Institute, King Saud University, P.O. Box 800, Riyadh 11421, Saudi Arabia; 2 Chemical Engineering Department, College of Engineering, King Saud University, P.O. Box 800, Riyadh 11421, Saudi Arabia; 3 Industrial Engineering Department, College of Engineering, King Saud University, P.O. Box 800, Riyadh 11421, Saudi Arabia; 4 Center of Excellence for Research in Engineering Materials (CEREM), Deanship of Scientific Research, King Saud University, P.O. Box 800, Riyadh 11421, Saudi Arabia

**Keywords:** biocomposite, polylactic acid, date palm fiber, extrusion, 3D printing, characterization

## Abstract

Biocomposites incorporating bio-based polymers and natural fibers hold great promise due to their environmental and economic benefits, though their commercial use is still limited by production challenges. This study reports the development of polylactic acid (PLA) composite filament reinforced with 5 wt% date palm fibers for fused deposition modeling (FDM)-based 3D Printing. The biocomposite is fabricated through extrusion and 3D Printing, and its mechanical, thermal, and water absorption properties are characterized in this work. Fiber dispersion is examined using a scanning electron microscope (SEM), while tensile testing evaluates yield strength, tensile strength, and elongation at break. Fracture behavior and failure mechanisms are further analyzed through optical microscopy and SEM. The biocomposite shows higher yield strength (36.75 MPa) and tensile strength (53.69 MPa), representing improvements of 10.12% and 6.53%, respectively, compared to in-house extruded pure PLA. However, it exhibits lower ductility, as indicated by reduced elongation at break. Water absorption is also higher in the biocomposite (0.58%) than in pure PLA (0.10%). Both materials display similar thermal behavior and brittle fracture characteristics. These results highlight the reinforcing effect of date palm fibers and the role of processing on the behavior/performance of the biocomposite. Reinforcing PLA with a small fraction of date palm fibers, an abundant natural resource, offers a cost-effective and eco-friendly material, particularly suited for single-use plastic products where biodegradability and sustainability are essential. This study also confirms the suitability of PLA/date palm fiber filament for FDM-based 3D Printing.

## 1. Introduction

Biocomposites, composed of biopolymers reinforced with natural fibers, have emerged as a sustainable alternative to non-biodegradable, petroleum-based materials, driven by environmental concerns, fossil resource depletion, and the need to reduce carbon emissions. Industries such as aerospace, energy, nuclear power, and construction are increasingly interested in the mechanical performance of these materials [1]. Their lightweight nature, adequate mechanical properties, low carbon footprint, energy efficiency, and cost-effectiveness make them highly attractive. Natural fiber-reinforced polymer composites combine the renewable advantages of plant-based fibers with the adaptable mechanical properties of biopolymer matrices [2]. Among biopolymers, polyhydroxyalkanoates, polylactic acid (PLA), and polybutylene succinate have shown significant potential. PLA, in particular, is the most widely used matrix due to its biodegradability, availability, environmental friendliness, antimicrobial nature, and attractive mechanical and thermal characteristics [3,4]. PLA-based biocomposites are not only sustainable but also offer a lightweight and affordable alternative to traditional synthetic materials.

Date palm trees are native to different locations, including the Middle East, and they produce a lot of biowaste that is not well-managed [5,6]. By producing affordable, eco-friendly materials, the vast volume of biowaste that is currently being wasted has the potential to support industrial sustainability. With more than 120 million date palm trees worldwide, including 80 million in the Arab world, date palm fibers are a plentiful natural resource [7]. Date palm fibers are abundant, compatible with polymers, and are ideal for sustainable biocomposites in industries. Their annual production surpasses coir by over 40 times, and hemp and sisal by 20 and 10 times, respectively [8]. When choosing natural fiber composites, fiber density is key since it lowers the composite’s weight, which makes it appropriate for sectors like aerospace and automotive. Date palm fibers have a density of 0.9 to 1.2 g/cm^3^, which makes them useful for military, furniture, sporting, and many other applications [9]. Because of their chemical structure, natural fibers readily absorb moisture, which results in weak spots in biocomposites that lower mechanical strength, cause fiber swelling and degradation, rupture, and make them more vulnerable to fungal attack [10]. However, date palm fibers perform relatively well; their water uptake is generally lower than many other common plant fibers [7]. Furthermore, compared to other natural fibers, date palm fibers are among the least expensive [7]. A biodegradable agricultural waste, date palm fiber, is becoming more and more popular as an environmentally friendly reinforcement for thermoplastic and thermoset composites [11,12]. Tensile strength, stiffness, elastic modulus, and flexural durability can all be improved by its high cellulose content and microfibril structure. Date palm fiber composites are utilized in cementitious composites, packaging, automobile interiors, and environmentally friendly building materials because they provide superior thermal stability and optimal conductivity for insulation.

Numerous studies from the literature pertaining to the production and characterization of date palm fiber-based biocomposites can be highlighted. The physical, thermal, and mechanical characteristics of date palm fiber-reinforced poly (β-hydroxybutyrate) composites produced through melt blending, compression molding, and annealing were examined by Mlhem et al. [13]. Investigations were conducted into the effects of date palm fiber loading (10–50 wt%) on the manufactured composites. Al Abdallah et al. [14] investigated the thermal insulation and mechanical properties of PLA biocomposites reinforced with date palm fiber. Prior to being reinforced in PLA with weight percentages ranging from 10 to 40 wt%, the fibers were treated using two distinct solutions. Biocomposite test specimens were manufactured by melt blending, molding, and annealing. Abu-Jdayil et al. [15] developed an environmentally friendly polymer composite as an insulating material using PLA and date palm wood powder. In a melt extruder, date palm powder (10–50 wt%) was combined with PLA, and then compression molding and annealing were performed. Physical, thermal, and mechanical properties of the composites were examined. In order to enhance biodegradability and increase renewability, Raza et al. [16] combined polystyrene and date palm fibers in varying weight percentages (10–40%) to produce a sustainable heat-insulating composite for building. Awad et al. [3] studied the physical and mechanical properties of PLA/date palm fiber biocomposites fabricated by melt-mixing and compression molding processing techniques. Four different fiber contents (10, 20, 30, and 40 wt%) were examined in this work. A bio-based and biodegradable film was manufactured by Kharrat et al. [17] by extrusion blowing of PLA-based thermoplastic resin compounded with natural fillers obtained from untreated date palm leaves. Tension tests were conducted along three directions on a pure PLA reference film that had no filler material and films with various filler contents. Tensile strength, Young’s modulus, and elongation at break were all improved by the ideal reinforcement content. Date palm fibers were also utilized to reinforce composites made of high-density polyethylene and polyvinyl chloride in the research by Maou et al. [18]. Composites with raw and chemically processed date palm fibers (loading of 30 wt%) were produced using a twin-screw extruder and compression molding. The study examined the composites’ morphological, thermal, mechanical, rheological, and water-absorption properties.

Natural-fiber-reinforced PLA biocomposites have garnered increasing attention owing to their environmental sustainability and economic viability. Numerous studies have focused on composites with relatively high fiber loadings (10–40 wt%) fabricated through melt-mixing and compression molding. However, despite their advantageous environmental attributes and regional abundance, date palm fibers remain underexplored as reinforcement in polymer composites. The application of 3D Printing, particularly fused deposition modeling (FDM), for fabricating natural-fiber-reinforced composites has been relatively limited, even though 3D Printing offers notable advantages such as reduced material wastage, minimal energy consumption, and lower carbon emissions. The limited adoption can be attributed to the challenges associated with producing dimensionally stable and printable filaments. Factors such as filament diameter inconsistency, fiber agglomeration, nozzle clogging, uneven extrusion flow, etc., often hinder successful fabrication via 3D Printing [19,20,21,22]. The controlled fiber content and appropriate selection of process parameters during both filament extrusion and 3D Printing are critical for achieving adequate fabrication quality and optimal performance of natural-fiber-reinforced biocomposites.

The fiber loading should not be increased without considering the capabilities of the filament maker and 3D Printer and ensuring that their appropriate process parameters are applied. Higher fiber contents lead to processing challenges in 3D printing, highlighting the importance of maintaining controlled fiber concentrations in polymer composites. For example, Srivastava et al. [23], reported that in hemp fiber-reinforced PLA composites, increased fiber content led to higher porosity, poor dispersion, weak interfacial bonding, and fiber clustering, thereby reducing material homogeneity. Similarly, Pereira et al. [19], found that filament containing 5 wt% fiber exhibited minimal diameter variation, smooth printing behavior, and no nozzle blockage, whereas 10 wt% loading resulted in frequent obstruction and fracture, and 15–20 wt% loading was unsuitable for 3D printing. The optimal fiber content for printability in natural-fiber-reinforced PLA composites is reported to range between 2% and 6%, as higher loadings often compromise print quality [23]. Therefore, although natural-fiber-reinforced PLA filament represents a promising research direction, further optimization is required to enhance processability and ensure consistent 3D Printing. It is also important to understand the effects of extrusion-based filament production and 3D printing on the mechanical, thermal, and water absorption properties of the composites. Furthermore, the performance of natural-fiber-reinforced filament should be compared with that of pure PLA filament processed under identical conditions.

The novelty of this work lies in integrating date palm fibers into 3D Printing, to avail their favorable properties and positive environmental impacts. It focuses on developing a date palm fiber-reinforced PLA biocomposite filament using an extrusion-based filament maker for FDM-based 3D Printing and analyzing its properties. The study identifies appropriate process parameters for both the filament maker as well as 3D Printing. This approach is novel, as the incorporation of natural fiber reinforcements, particularly date palm fiber at low loadings (5 wt%), into extruded filament for 3D Printing has received limited attention in recent years.

The present work aims to fabricate and characterize PLA/date palm fiber biocomposite with 5 wt% fiber content. The key objectives are to: (1) produce biocomposite filament with an extrusion-based filament maker and fabricate specimens using 3D Printing; (2) evaluate thermal transitions and crystallinity changes induced by date palm fiber inclusion using Differential Scanning Calorimetry (DSC); (3) assess tensile strength, Young’s modulus, and elongation at break using standardized tensile tests; (4) examine water uptake behavior; and (5) analyze fracture mechanisms through optical microscopy and scanning electron microscope (SEM). This study benchmarks the properties of PLA/date palm fiber biocomposite against an in-house extruded pure PLA filament to demonstrate the reinforcing effect of low fiber loadings and their potential for sustainable and eco-friendly applications.

## 2. Materials and Methods

**Figure 1:**
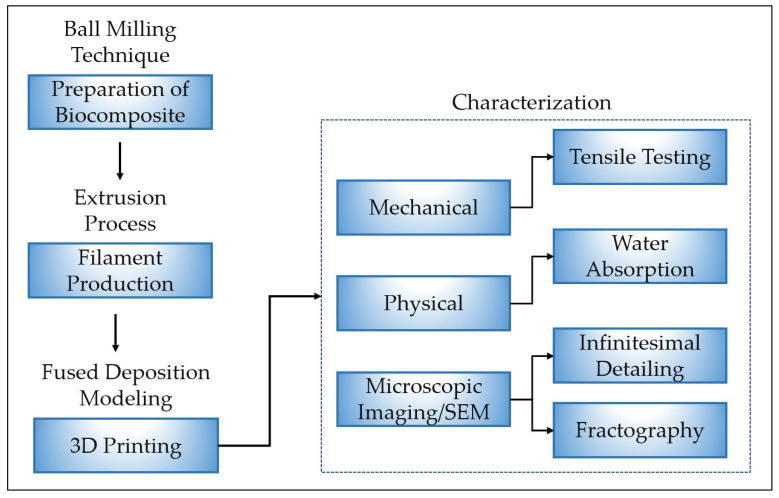
Methodology adopted in this study.

This section outlines the materials and methodology employed in this study. The research methodology follows four key stages: preparation of the date palm fiber-PLA biocomposite, production of the biocomposite filament, fabrication of standard specimens for tensile test and water absorption study, and mechanical and physical characterization of the composite material (Figure 1).

### 2.1. Preparation of Biocomposite

**Figure 2:**
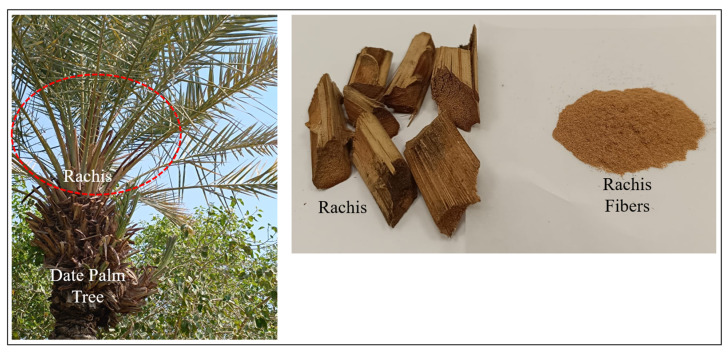
Rachis from the Date Palm Tree.

The biocomposite developed in this study consists of PLA reinforced with natural date palm fiber. The fiber is sourced from the rachis of date palm trees (refer to Figure 2), which is manually separated from the tree and then cut into shorter segments. Date palm fiber typically comprises 43–46 wt% cellulose, 18–24 wt% hemicellulose, 20–28 wt% lignin, 5–10 wt% ash, and 2–11 wt% moisture content [24,25,26,27].

**Figure 3:**
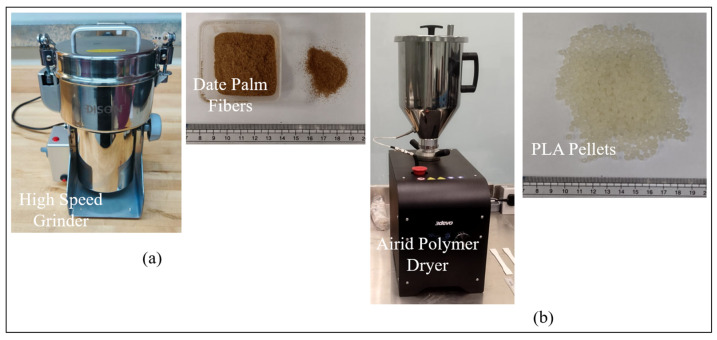
(**a**) High-speed grinder and date palm fiber; (**b**) Airid polymer dryer and Pure PLA pellets.

To prepare the fiber for composite fabrication, it is thoroughly washed with distilled water and oven-dried at 60 °C for 24 h. The dried fiber is then ground using a high-speed grinder, having a speed of around 20,000–25,000 rpm (Figure 3a). The resulting material is sieved through mesh sizes of 150 µm and 180 µm to ensure uniformity in particle size. The process of grinding and sieving is repeated continuously until fibers of the desired size are achieved, and the processed fiber is stored in a sealed container to maintain its dryness and prevent contamination (Figure 3a). The PLA used in this study is in the form of pellets, sourced from 3devo (3devo B.V., Utrecht, The Netherlands). These pellets are dried using an AIRID dryer (3devo, Utrecht, The Netherlands) at 45 °C for 3 h prior to use, as shown in Figure 3b.

**Figure 4:**
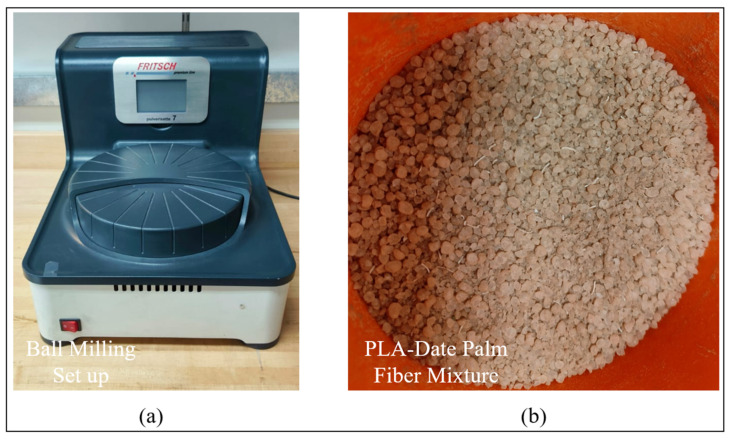
(**a**) Ball milling setup; (**b**) Mixture comprising PLA pellets embedded with date palm fiber.

PLA pellets are first ground into fine particles using a high-speed grinder. A composite blend is then prepared using this ground PLA and mixing it with 5 wt% of date palm fiber (150 to 180 μm in size). This mixture is subjected to mechanical alloying through high-energy ball milling using Pulverisette P7 (Fritsch, Markt Einersheim, Germany) as shown in Figure 4a. The milling process is carried out in an airtight cylindrical stainless-steel container. The fine powders intended for mixing are introduced alongside two stainless steel balls to aid in the mixing process. The mixing process is conducted at a rotational speed of 200 rpm for a duration of 1 h. To ensure effective cooling and prevent temperature accumulation within the container, the process is momentarily paused for 5 min after every 15 min of mixing. This milling process which involves repeated fracturing and cold welding of particles helps achieve a uniform distribution of date palm fiber within the PLA matrix. The resultant composite powder consisting of PLA with well-dispersed natural fiber (refer to Figure 4b) is now suitable for subsequent processing.

### 2.2. Filament Extrusion

**Figure 5:**
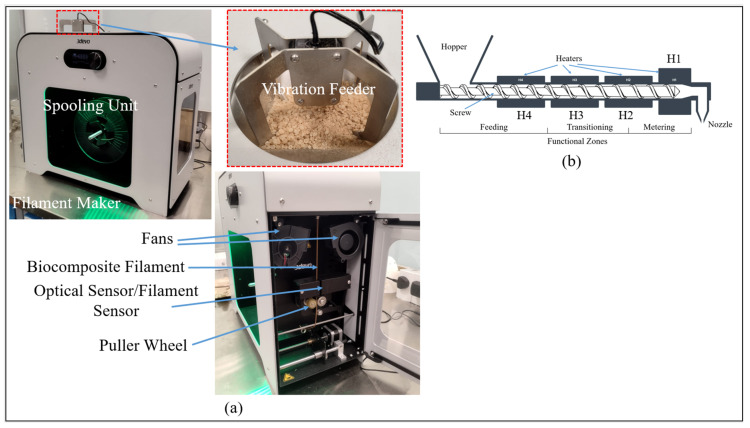
(**a**) Filament extrusion system; (**b**) Functional zones within the extrusion system (Reproduced with permission from 3devo B.V., Utrecht, The Netherlands).

Filament extrusion is carried out using the 3devo Composer 350 filament maker (3devo B.V., Utrecht, The Netherlands) as shown in Figure 5a. It is a compact system featuring a single-screw extruder, multiple independently controlled heating zones, a dual-fan cooling system, an optical sensor for real-time diameter monitoring, and an integrated spooling unit [28]. The biocomposite mixture prepared earlier is fed into the extruder using a vibration feeder, which ensures consistent material flow. The feeder prevents common issues such as agglomeration, clogging, and flow disruptions, often associated with coarse and irregular particles, by breaking up cohesive structures within the hopper. This promotes a smoother and more stable extrusion process, resulting in uniform filament quality. The feeder operates with one-second pulse intervals, gently vibrating the mixture to maintain a steady feed into the extruder. The extruder is equipped with a specially designed single screw mechanism that facilitates thorough mixing of date palm fiber and PLA before extrusion. This ensures a well-blended output and consistent filament formation. The extruder comprises three functional zones: the feeding zone, the transition zone, and the metering zone, as shown in Figure 5b. Four heaters are strategically positioned across these zones, and their temperature settings are carefully tuned based on the material properties to achieve optimal processing conditions.

Before initiating filament extrusion, essential operational parameters such as the temperature settings for each heating zone, screw rotation speed, cooling fan speed, and spool dimensions are configured within the system. Accurate measurements, including the spool’s width and its full and empty diameters, are entered to ensure consistent tension during the winding process [28]. Furthermore, the filament maker (extruder) maintains precise control of the filament diameter by continuously monitoring it with an optical sensor and adjusting the winding speed as needed.

The filament extrusion of the date palm fiber (5 wt%) reinforced PLA biocomposite is initiated using the extrusion parameters obtained for pure PLA, since the PLA constitutes 95% of the composite. However, the output filament diameter remains consistently low, in the range of 1.55–1.65 mm, which is below the desired 1.75 mm. This reduction is attributed to insufficient material flow through the barrel, resulting in less material being extruded. Since the biocomposite consists of both PLA and date palm fibers, the feedstock tends to form lumps, cohesive structures, and agglomerates at the hopper. To address this, a feeder is employed at the hopper, as shown in Figure 5a, to ensure smooth and uniform feeding of the material into the barrel. Despite this adjustment, the output filament diameter continues to deviate from the target, indicating that the problem of insufficient material flow persists.

To improve material flow, the screw speed is increased from 5 rpm (for pure PLA) to 6.5 rpm. Nevertheless, the issue remains, with the filament diameter still below the desired 1.75 mm. This outcome suggests that the material flow through the barrel is still inadequate. However, further increasing the screw speed is not recommended. As reported in [29], optimum results are generally achieved when the screw speed lies between 2 rpm and 8 rpm. At higher screw speeds, the material spends less time in the barrel, absorbs less heat, and may remain partially unmelted, leading to incomplete melting. Excessive screw speed may also cause clogging in the screw or nozzle, poor homogenization of the melt, and difficulty for the puller mechanism to maintain a consistent filament diameter [30]. Adequate residence time in the barrel is also required to ensure uniform melting, which further explains why the screw speed cannot be increased significantly.

The screw speed is thus fixed at 6.5 rpm, and the focus shifts to tuning the heater temperatures to enhance melting and material flow. The extrusion system operates with four heaters that define the temperature profile across the feed, transition, and metering zones. In this study, only the heaters in the transition zone are adjusted, while the feeding and metering zone heaters remain at their initial values set for pure PLA. The feeding zone (H4) is maintained at 170 °C to avoid premature melting, as early melting reduces pressure buildup inside the barrel. Pressure buildup is essential for stable extrusion and high-quality filament formation. Heater 4, located closest to the hopper, initiates the melting process, but solid material in this region is also necessary to push the feedstock forward effectively [31]. Allowing the material to melt further downstream promotes better pressure buildup and a more consistent output filament.

The optimization process therefore involves first increasing the screw speed from 5 rpm to 6.5 rpm, followed by incremental adjustments to the two transition zone heaters (Heaters 3 and 2). This section of the barrel, also known as the compression zone, is responsible for transforming the material into a fully molten and homogeneous state. Beyond Heater 2, the material is expected to be completely melted [31]. In this study, the temperatures of Heater 2 (H2) and Heater 3 (H3) are increased in increments of 1 °C. This approach ensures both sufficient pressure buildup in the barrel and complete melting of the material. Since Heater 3 is located nearer to the feed zone, its temperature should be maintained to prevent excessive heating. By contrast, Heater 2 temperature, located near the metering zone, is raised to guarantee full melting before the material enters the metering section. In addition, friction in the compression zone contributes extra heat; so the temperatures in the transition zone should be controlled so it does not lead to overheating and undesirable melting behavior. With the adopted strategy, the material undergoes a gradual and controlled melting process, neither too rapid nor too delayed, ensuring stable pressure buildup and consistent filament diameter.

The fan cooling speed is maintained at 30% for both pure PLA and the biocomposite, as the filament maker is operated in a relatively cold room. At this setting, the filament diameter remains consistent and within the desired range. Maintaining the proper cooling speed is crucial, since excessive cooling makes the filament too rigid and difficult to control, preventing it from being pulled to the correct thickness [30]. The parameter settings thus obtained through the above-described iterative process result in a stable filament diameter of 1.75 ± 0.05 mm. The final extrusion parameters used for producing the biocomposite filament are summarized in Table 1. For comparison, pure PLA filament is also fabricated using the same PLA pellets employed in the biocomposite mixture. This allows for a direct performance evaluation between the pure PLA and the date palm fiber reinforced PLA biocomposite.

### 2.3. Thermal Characterization

**Figure 6:**
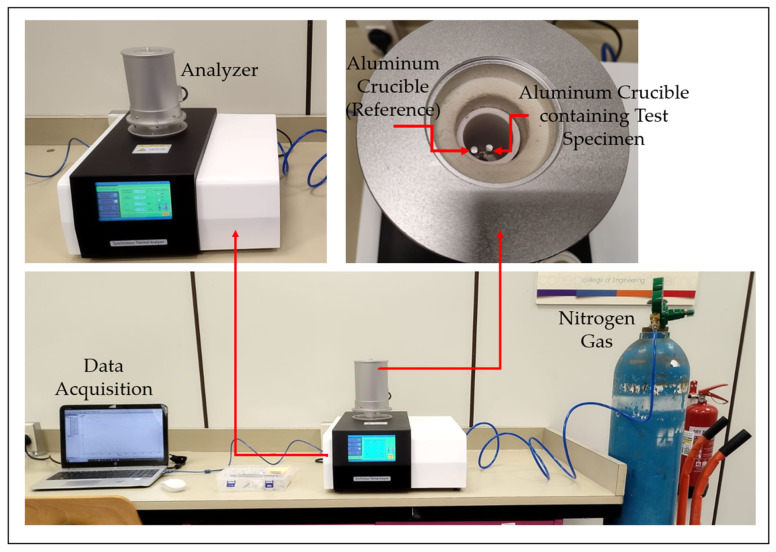
Differential scanning calorimetry setup.

Prior to 3D Printing, the thermal properties of both the biocomposite and pure PLA were characterized using DSC. The DSC analysis is used to determine the thermal properties, including the glass transition temperature (T_g_), cold crystallization temperature (T_c_), and melting temperature (T_m_). The test uses biocomposite and pure PLA filaments produced in the previous step, with each sample weighing approximately 10 mg. The analysis is conducted using an LR-STA200 Synchronous Thermal Analyzer (Lonory, Dongguan, China) in accordance with ASTM D3418 standard [32,33,34]. As shown in Figure 6, the specimens are placed in aluminum crucibles, and the initial temperature is set to ambient conditions, approximately 20 to 25 °C. The temperature is then increased at a constant rate of 10 °C/min until it reaches the final temperature, which varies depending on the material. For pure PLA, the end temperature is set at 220 °C. The same temperature is applied to the biocomposite, as it consists of 95% PLA. This temperature limit is selected based on supplier data. Throughout the test, nitrogen gas flows into the chamber at a rate of 50 mL/min to prevent contamination.

### 2.4. Three-Dimensional Printing

**Figure 7:**
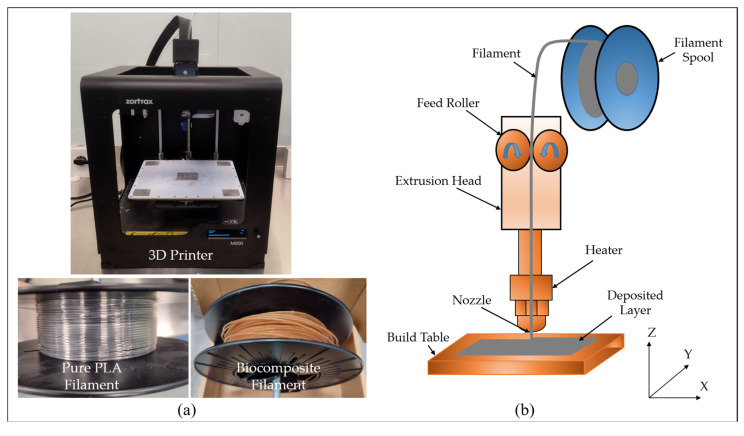
(**a**) Zortrax M200 3D printer; (**b**) Illustration of FDM [38].

3D Printing is performed using a tabletop FDM-based 3D printer, the Zortrax M200 (Zortrax, Olsztyn, Poland), as shown in Figure 7a. FDM is a widely used additive manufacturing process for fabricating 3D-printed polymers [35]. This process involves the extrusion of material in the form of a fine filament through a heated nozzle. The extruded material is deposited along a predefined path onto the build platform, where it rapidly solidifies. Successive layers are subsequently deposited and fused with the underlying layers, enabling the fabrication of 3D structures. In this study, a Zortrax M200 printer is employed, utilizing a 1.75 mm diameter filament and providing a build volume of 200 × 200 × 180 mm. A 0.4 mm diameter nozzle is employed for extrusion. The printer is equipped with a single extruder and a heated build platform [36]. The maximum achievable extruder temperature is 290 °C, while the platform can reach up to 105 °C. The system operates with a power consumption of 200 W [37] and is compatible with Z-SUITE slicing software. Figure 7b illustrates the schematic of the FDM process and the working principle of the Zortrax M200. The extrusion head, driven by an electric motor, receives the filament from a spool. The build platform moves along the Z-axis, while the extrusion head travels in the X and Y directions to deposit material.

The FDM process involves two key stages: pre-processing and manufacturing. In the pre-processing stage, the specimen is designed using computer-aided design (CAD) software (V5) and exported in standard tessellation language (STL) format. The STL file is then imported into Z-SUITE software (Version 2.32.0.0, Zortrax, Olsztyn, Poland), where G-code instructions for the printer are generated. All relevant process parameters, including bed and extrusion temperatures, layer height, print speed, and infill percentage, are defined before slicing the model. In this study, parameter settings are optimized based on the filament material to ensure high-quality prints.

The specimens made of pure PLA are fabricated successfully using standard 3D Printing parameters, namely an extrusion temperature of 210 °C, a build platform temperature of 55 °C, and a print speed of 70 mm/s. Initially, the same process parameters are applied to the PLA biocomposite; however, the material fails to extrude from the nozzle. This issue arises because the extrusion temperature is too low for the biocomposite, resulting in incomplete melting of the filament and insufficient material flow. An extrusion temperature that is too low causes under-extrusion, clogging, or incomplete extrusion, as the filament does not melt adequately to pass smoothly through the nozzle.

A slight increase in extrusion temperature from 210 °C to 215 °C improves the flow but does not resolve the problem completely, while a further increase to 220 °C enhances material flow but remains insufficient. Increasing the extrusion temperature beyond this range is avoided, as excessively high temperatures can make the material overly fluid, cause partial thermal degradation, or even induce crosslinking and burning of the filament [39,40,41]. Therefore, instead of raising the extrusion temperature further, the print speed is adjusted. Reducing the print speed first by 5% and then by 10% improves the extrusion quality, with consistent and smooth material flow achieved at 63 mm/s. This observation aligns with the report of [42], which notes that high-speed printing hinders continuous material flow, produces thinner deposited layers, and weakens interlayer bonding. Lower printing speeds are also advantageous because they increase the residence time of the filament in the nozzle, ensuring sufficient melting and reducing the risk of clogging.

Once satisfactory material flow is established through the combination of higher extrusion temperature and reduced print speed, another issue is observed: inadequate adhesion between the first layer and the build platform, accompanied by warping. To mitigate this, the build platform temperature is raised from 55 °C to 65 °C, which promotes better adhesion. A similar approach is reported by Srivastava et al. [23], who successfully 3D-printed 2.5% hemp fiber-reinforced PLA by increasing the nozzle temperature from 200 °C to 210 °C, reducing the print speed from 45 mm/s to 40 mm/s, and raising the bed temperature from 60 °C to 65 °C. Their optimization strategy for fiber-reinforced PLA closely corresponds to the adjustments employed in this study.

Table 2 presents the specific printing parameters for both the date palm fiber-PLA biocomposite and pure PLA.

**Figure 8:**
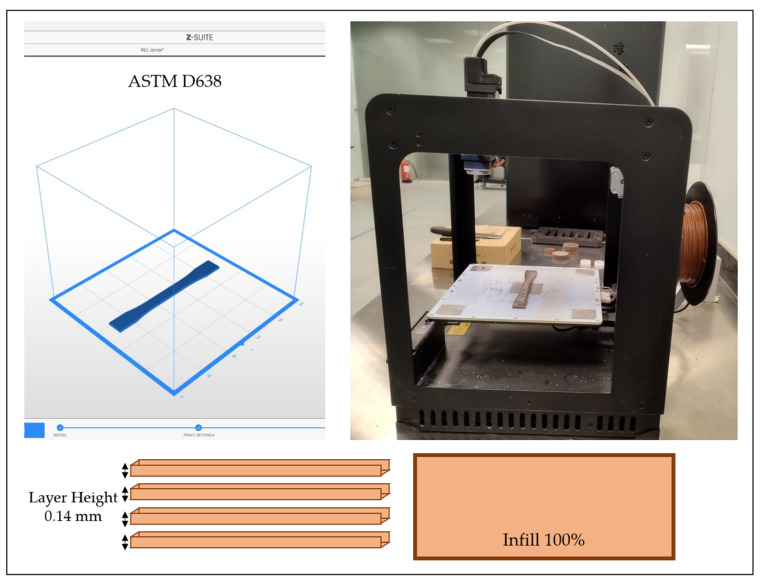
Description of printing parameters.

Specimens are printed flatwise along the X-axis, as shown in Figure 8. The infill percentage is maintained at 100%, and the layer height is set at 0.14 mm. Three samples of each material are printed and tested to ensure consistency and eliminate bias. The average of the three measurements is used for the final evaluation. During the manufacturing stage, the filament is heated to a semi-molten state and extruded layer-by-layer to fabricate the object. Two-dimensional (2D) layers are deposited on the build platform and stacked to create the 3D object. After each layer is deposited, the platform moves downward, and the extrusion process continues until the final part is completed.

### 2.5. Characterization of Mechanical and Physical Properties

**Figure 9:**
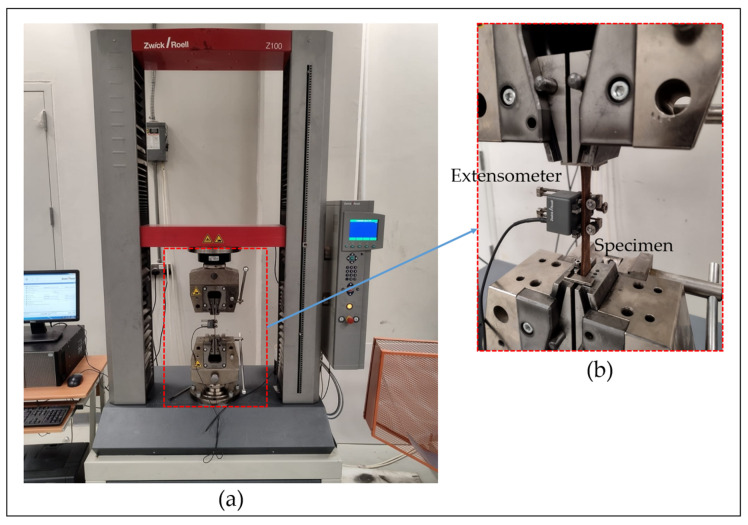
(**a**) Universal tensile testing machine; (**b**) Enlarged view of the setup.

The mechanical properties of the materials are evaluated through tensile testing, carried out in accordance with ASTM standards. Specifically, the ASTM D638 standard is followed for testing polymer specimens [43,44]. As illustrated in Figure 9a, a Zwick Z100 electromechanical universal testing machine (ZwickRoell, Ulm, Germany) equipped with a 100 kN load cell is used to measure tensile strength, yield strength, and elongation at break. An extensometer, shown in Figure 9b, is attached to the specimen to accurately measure strain within the elastic region. The yield point, which represents the transition from elastic to plastic deformation, is determined using the 0.2% strain offset method [45]. The corresponding stress value at this offset is reported as the yield strength. Tensile testing is performed at a constant crosshead speed of 5 mm/min, as specified for Type I specimens under the ASTM D638 standard [43,46,47].

**Figure 10:**
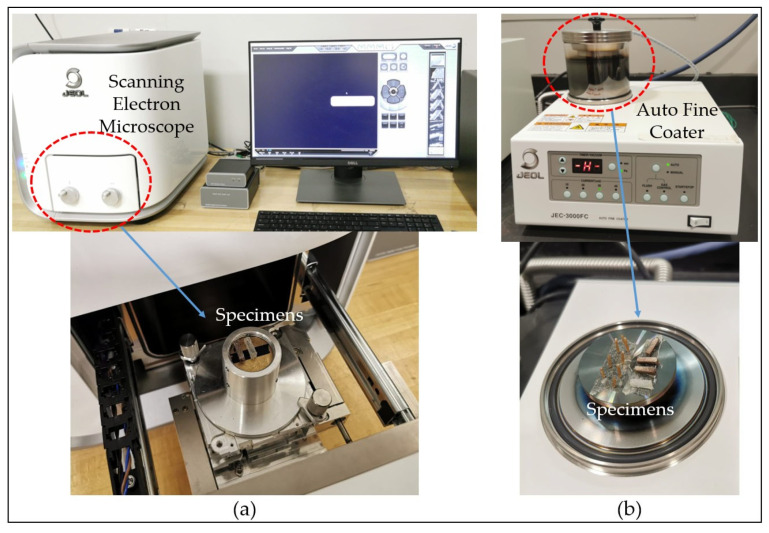
(**a**) SEM setup; (**b**) Coating Machine.

To investigate reinforcement dispersion, the fiber distribution within the PLA matrix is examined using a SEM (JEOL JCM-6000 Plus, JEOL, Tokyo, Japan), as shown in Figure 10a. A cross-sectional SEM image of the FDM filament is obtained by immersing the biocomposite filament in liquid nitrogen for 10 min, followed by brittle fracturing. The resulting fracture surface is coated with a thin layer of platinum to enhance conductivity before SEM imaging. The coating is performed using an auto fine coater (JEOL JEC-3000 FC, JEOL, Tokyo, Japan) as shown in Figure 10b.

**Figure 11:**
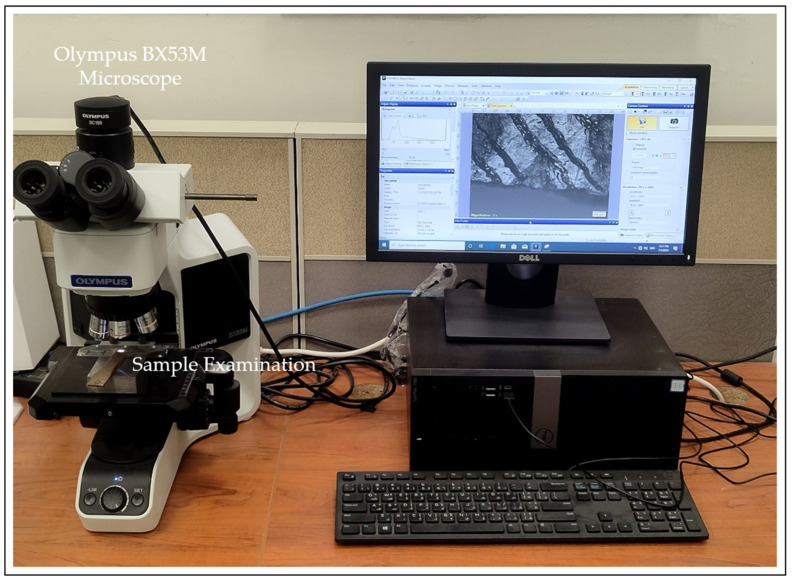
Optical microscope setup for infinitesimal detailing.

The fabricated specimens are also examined using an optical microscope to identify the specific fracture modes. An Olympus BX53M upright optical microscope (Olympus, Tokyo, Japan) is used for this purpose, enabling detailed microscopic observation. As shown in Figure 11, the microscope captures high-resolution images using a 100× objective lens, with illumination provided from below to enhance image clarity [48,49]. In this study, images are captured at 5× magnification. In addition, high-magnification SEM analysis is performed to study the failure mechanisms during tensile testing, with the fractured surfaces coated with platinum before imaging.

This study also examines the water absorption behavior of the biocomposite to evaluate its resistance to moisture. The analysis is conducted in accordance with the ASTM D570-98 standard [50]. Disk-shaped samples (50.8 mm × 3.2 mm) of pure PLA and the biocomposite (three each) are fabricated using 3D Printing [50,51]. The samples are dried, weighed (W_i_), and immersed in distilled water at room temperature for a period of 24 h, similar to the procedure adopted by Awad et al. [3] for PLA reinforced with date palm fiber. After 24 h of immersion, the samples are removed, gently wiped with clean tissue to eliminate excess surface water, and reweighed (W_f_). The percentage of water absorption is calculated using Equation (1) [19,51].

(1) $$\mathrm{Water}\;\mathrm{absorption}\;(\%)=\frac{W_{f}-W_{i}}{W_{i}}\times 100$$

where W_i_ represents the initial weight before immersion and W_f_ denotes the final weight after immersion.

**Figure 12:**
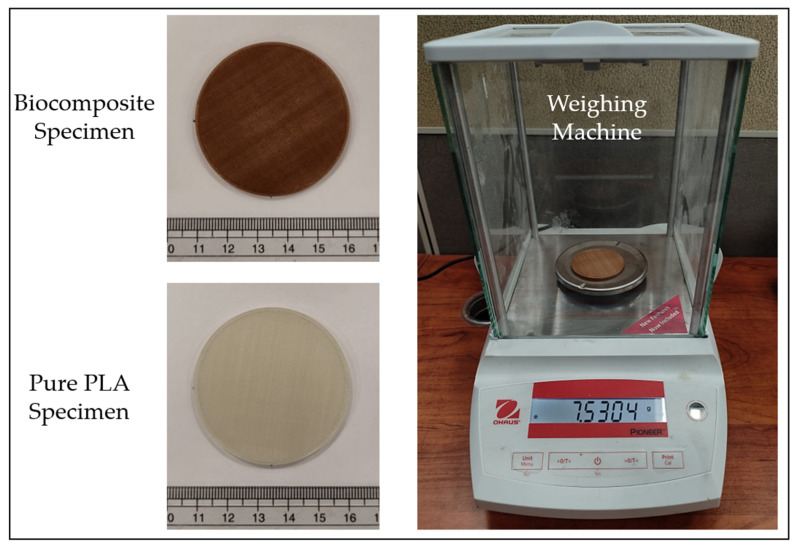
Three-dimensional-printed samples and weighing machine for water absorption study.

The samples used in the water absorption study are shown in Figure 12.

## 3. Results and Discussion

**Figure 13:**
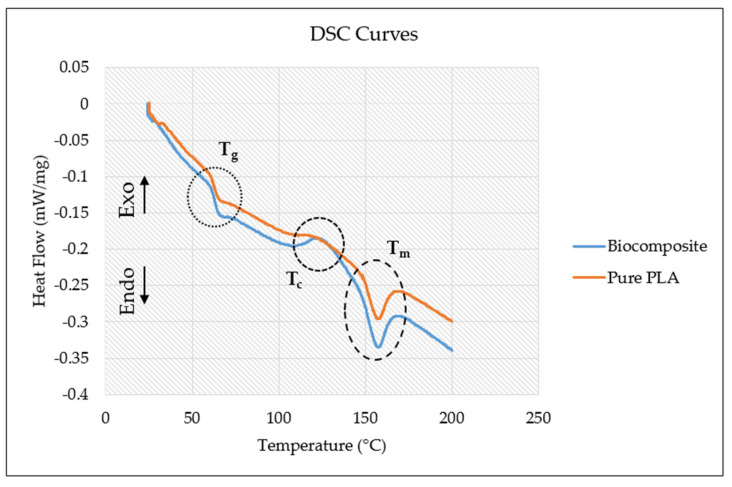
DSC curves for the biocomposite and pure PLA.

Figure 13 presents the DSC curves of pure PLA and the PLA/date palm fiber biocomposite filaments. Both materials exhibit the typical thermal transition behavior, characterized by three distinct processes: the glass transition (T_g_), an exothermic cold crystallization peak (T_c_), and an endothermic melting peak (T_m_). The DSC results for T_g_, T_c_, and T_m_ of the filaments are summarized in Table 3. A comparable DSC curve for PLA, similar to that obtained in this study, has also been reported in [52]. In the present study, both pure PLA and the PLA/date palm fiber biocomposite display comparable T_g_ and T_m_ values, indicating that the fundamental thermal transitions remain largely unaffected due to fiber reinforcement. The glass transition occurs at approximately 63 °C in both samples, evidenced by a distinct change in the heat flow slope, marking the transition from a rigid glassy state to a rubbery state [53].

A notable difference, however, is observed in the cold crystallization behavior. In pure PLA, the cold crystallization peak is less pronounced, whereas in the biocomposite it is more distinct, reflecting the influence of the date palm fibers. The biocomposite exhibits a clear exothermic peak at around 124.1 °C, which is less apparent in pure PLA. This behavior can be attributed to the nucleating effect of the date palm fibers, which promote heterogeneous crystallization and enhance the overall crystallinity of the polymer matrix. Similar findings have been reported by Cao et al. [54] and Saleh et al. [55], who observed cold crystallization peaks in polymer composites containing reinforcing agents, while such peaks were barely visible in the corresponding pure polymers. The influence of natural fibers on enhancing the crystallinity of polymer matrices has been consistently documented in the literature [56,57]. Additionally, interactions between polymer chains and fiber surfaces can induce transcrystallinity, thereby improving fiber-matrix adhesion [57,58]. These structural modifications enhance stress transfer efficiency at the interface, which helps explain the improved mechanical performance of the biocomposite despite the absence of significant differences in T_g_ and T_m_.

Both materials also display a second endothermic peak (representing melting temperatures) at approximately 156.9 °C, consistent with previously reported values [59,60]. While the overall thermal transitions are similar, the biocomposite shows a slightly deeper heat flow, suggesting higher thermal activity, likely due to fiber-induced crystallization effects. Overall, these results indicate that date palm fibers subtly modify the thermal response of PLA while contributing to its enhanced mechanical properties.

**Figure 14:**
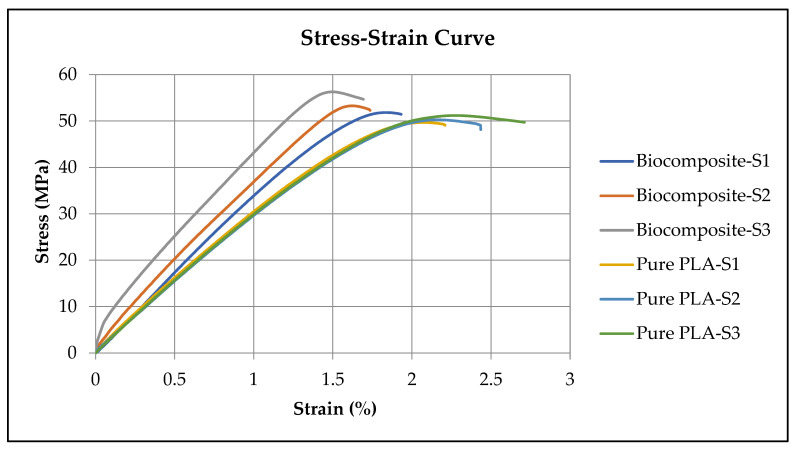
Stress–strain curves for three specimens each of the biocomposite and pure PLA.

Figure 14 illustrates the stress–strain curves for both biocomposite and pure PLA samples under tensile loading. The curve for pure PLA reveals a small region of plastic deformation beginning around 2% strain, with an average elongation at break of approximately 2.45%. In comparison, the biocomposite also exhibits an initially linear elastic region followed by a brief plastic region. However, it fractures at a lower strain of about 1.93%, indicating reduced ductility. Although the biocomposite demonstrates higher tensile strength compared to pure PLA, it fails earlier, exhibiting a more brittle behavior. The transition from elastic to plastic deformation is subtle in both materials, with no clearly defined yield point observed in either case. This lack of noticeable yielding reflects their inherently brittle and rigid nature. The limited plastic deformation in both materials suggests high stiffness and a tendency to fracture suddenly under stress, rather than deform gradually. Notably, the biocomposite is more brittle than pure PLA, making it more susceptible to abrupt failure upon loading. This comparative analysis confirms that although both materials are brittle, pure PLA offers slightly better ductility and is marginally less prone to sudden fracture. Although the biocomposite exhibits a lower elongation at break, indicating reduced ductility, it outperforms pure PLA in terms of both yield and tensile strengths. The yield strength of the biocomposite is around 36.75 MPa, compared to 33.37 MPa for pure PLA, representing a 10.12% increase. Similarly, the tensile strength of the biocomposite reaches approximately 53.69 MPa, surpassing the 50.40 MPa of pure PLA by 6.53%. These improvements in mechanical strength are attributed to the reinforcement effect of incorporating 5 wt% of date palm fiber into the PLA matrix. The findings of this study align well with those reported in the literature. For instance, Fouly et al. [61] demonstrated that the addition of date pit particles to PLA reduced flexibility while enhancing its compressive strength. Similarly, Dhakal et al. [11] have reported improvements in tensile strength, modulus, and flexural properties when date palm fibers were incorporated into polymer composites. Neoh et al. [62] observed that natural fiber-reinforced composites consistently exhibited enhanced tensile strength. Ghanmi et al. [63] also noted mechanical property improvements with the addition of date palm fiber. Ebrahim et al. [64] identified date palm waste as a promising, low-cost, and biodegradable reinforcement for polymer composites. They emphasized that incorporating date palm waste can improve mechanical performance, thermal stability, and biodegradability, making them suitable for eco-friendly and sustainable applications.

**Figure 15:**
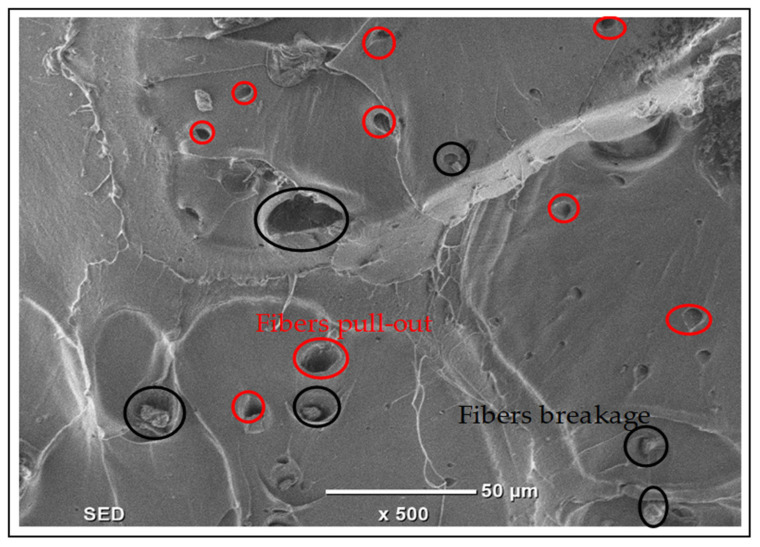
SEM cross-sectional view of PLA/date palm fiber biocomposite filament.

The SEM cross-sectional image of the PLA/date palm fiber biocomposite FDM filament (refer to Figure 15) reveals a uniform fiber distribution within the PLA matrix, evidenced by fiber pull-out voids (red circles) and fiber breakages (black circles).

**Figure 16:**
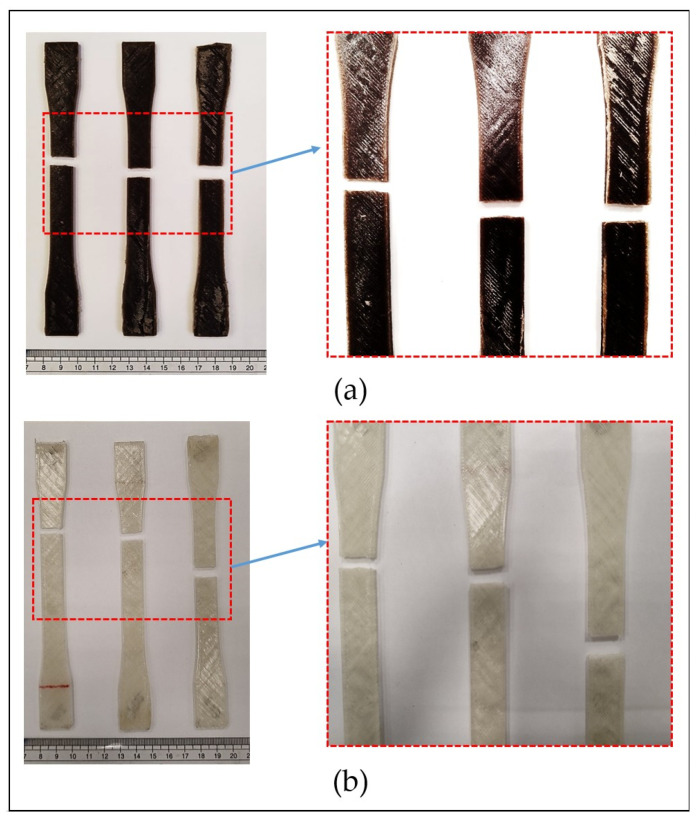
Fracture locations in (**a**) Biocomposite; (**b**) Pure PLA.

As shown in Figure 16a, the fracture analysis of the biocomposite samples reveals that failure consistently occurs near the centroid during tensile testing, indicating a predominantly centralized fracture mechanism. This suggests that the stress is uniformly distributed across the cross-sectional area of the specimens. Once the applied stress exceeds the material’s tensile strength, failure occurs abruptly, reflecting a brittle fracture behavior. The consistent fracture location also indicates an absence of significant localized defects or stress concentrations within the biocomposite structure. In contrast, Figure 16b shows that the pure PLA specimens generally fracture slightly above the central region, near the upper grip, with one exception where the fracture occurs at the midpoint. This irregular fracture pattern suggests the presence of material inconsistencies or pre-existing flaws in the PLA, which may serve as initiation sites for failure. Such behavior implies non-uniform stress distribution and highlights the potential presence of voids or microcracks in pure PLA that contribute to premature fracture at localized weak points.

It is also essential to understand the possible failure mechanisms. Previous studies on tensile failure have identified two main modes: interlayer and inlayer fractures [65]. To accurately interpret the tensile test results, both failure types must be characterized [66]. Interlayer failure occurs when adjacent layers separate at their interface without fully detaching, with a failure surface parallel to the layers (0° angle). In contrast, inlayer failure involves a fracture surface at a nonzero angle to the material layers, indicating failure within the layers themselves.

**Figure 17:**
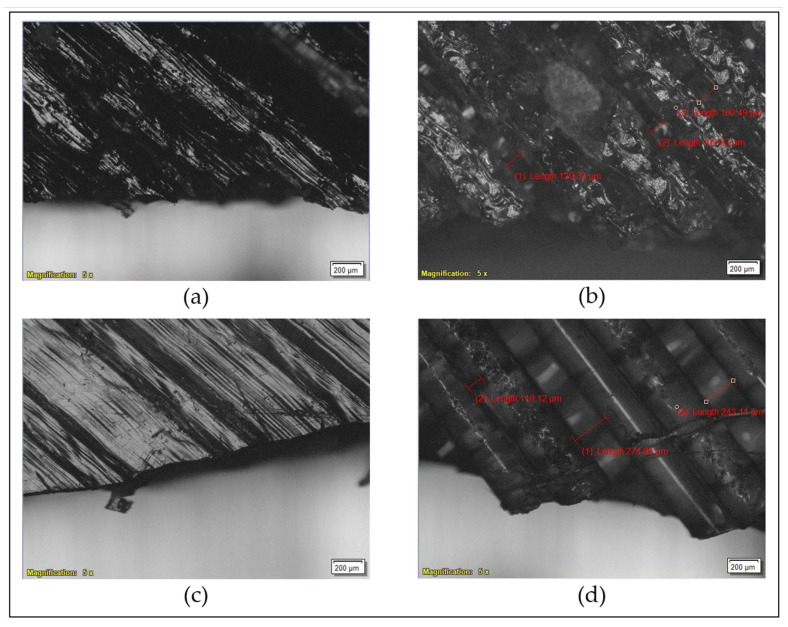
Microscopic examination of fracture surfaces: (**a**) Fracture morphology of the biocomposite; (**b**) Measured spacing between adjacent rasters in the biocomposite; (**c**) Fracture morphology of pure PLA; (**d**) Measured spacing between adjacent rasters in pure PLA.

The fracture profiles of the biocomposite and pure PLA, as shown in Figure 17a,c, indicate brittle failure with minimal ductility. The fractured surfaces exhibit smooth and flat regions with no evidence of plastic deformation. The direction of the applied tensile force is nearly perpendicular to the fracture plane, and the nonzero angle between the fracture surface and the raster direction confirms a predominantly inlayer failure mechanism in both materials. This suggests that failure is governed more by the intrinsic strength of individual rasters or fibrils within a layer than by the interlayer bonding between adjacent rasters. Microscopic observations further support this, particularly in the biocomposite, where fracture occurs primarily due to the breaking of individual rasters, with little evidence of delamination between layers. As shown in Figure 17b, the average spacing between adjacent rasters in the fracture zone of the biocomposite is approximately 133.22 µm, indicating tight raster packing. In contrast, Figure 17d reveals that pure PLA exhibits a greater separation between neighboring rasters near the fracture zone, with an average spacing of about 212.41 µm. While inlayer fracture remains the dominant mechanism in pure PLA, the presence of slightly larger gaps and signs of delamination suggest that interlayer debonding might have also contributed to its overall failure.

**Figure 18:**
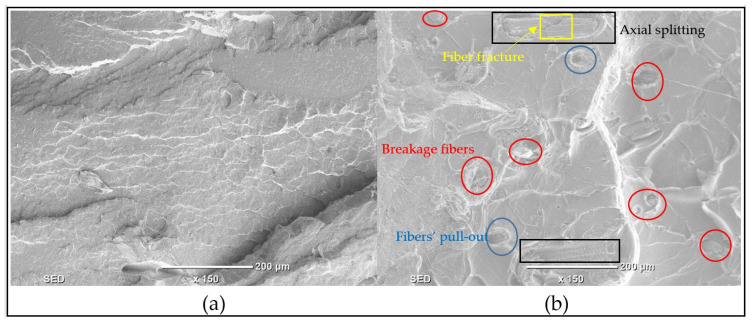
SEM images showing the fractography of (**a**) Pure PLA and (**b**) PLA/date palm fiber biocomposite.

Figure 18 presents the fractography of tensile specimens of PLA and PLA/date palm fiber biocomposite. The SEM image of pure PLA (refer to Figure 18a) shows a characteristic brittle fracture surface, with smooth and flat regions containing distinct river markings. These features confirm the inherent brittle nature of pure PLA, consistent with previous reports [67]. Cabrera et al. [68] similarly observed no evidence of plastic deformation including shear bands, necking, or whitening in PLA and PLA/Jute fabric composites, thereby confirming their brittle fracture behavior.

The PLA/date palm fiber biocomposite exhibits a similar fracture morphology, with smooth, flat fracture surfaces and river markings indicating brittle failure. However, failure mechanisms introduced by the presence of fibers are also evident, including fiber pull-out, fiber breakage, fiber fracture, and axial splitting (refer to Figure 18b). Among these, fiber breakage and fiber pull-out are the dominant modes of failures. A high degree of fiber breakage is typically associated with brittle fracture, as also reported in [69], where fiber cracking and fragmentation with slight debonding or pull-out resulted in a brittle failure of kenaf fiber-reinforced PLA composites. Fiber breakage, observed as transverse failure of the fibers, represents a critical failure mode under tensile stress, leading to a loss of load-bearing capacity in the composite [70]. Moreover, in some regions (refer to SEM image in Figure 18b), fibers exhibit fracture (cracking without full separation), while others show axial splitting, where fibers fail longitudinally along their axis rather than perpendicular to it. Instances of fiber pull-out are also observed, which occurs due to weak bonding between the fiber and the PLA matrix [71]. This issue can be reduced by applying surface treatments to the fibers. The occurrence of fiber breakage also suggests inlayer failure within the fiber-matrix structure of the biocomposite. The absence of layer separation or delamination in the SEM images further indicates that the failure occurs within the layers, characterized by brittle fracture along the fiber plane. The SEM investigation thus confirms that both pure PLA and the PLA/date palm fiber biocomposite exhibit brittle failure. Moreover, the presence of fiber breakage indicates that the failure mechanism is primarily inlayer dominant.

**Figure 19:**
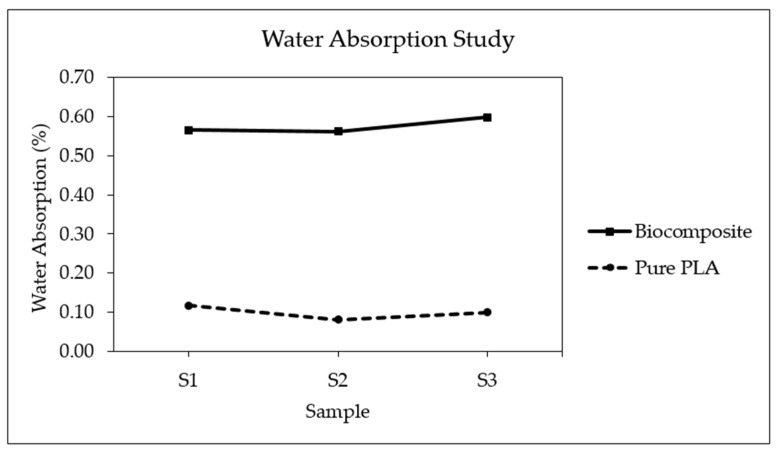
Propensity of water absorption for biocomposite and pure PLA.

Polymers are susceptible to degradation over time as a result of water absorption [72,73,74]. This moisture uptake can significantly impair their mechanical performance, leading to reductions in strength, stiffness, and long-term durability. Additionally, the presence of absorbed water may facilitate the growth of microorganisms, potentially resulting in bio-contamination by bacteria or fungi [75]. Understanding the water absorption characteristics of 3D-printed polymer materials is significant for assessing their reliability in moisture-exposed environments. As shown in Figure 19, the biocomposite containing date palm fiber exhibits a higher tendency to absorb water as compared to pure PLA. On average, the water absorption of the biocomposite is about 0.58%, whereas pure PLA absorbs only about 0.10%. For instance, Awad et al. [3] reported that PLA reinforced with 10 wt% date palm fiber absorbed approximately 0.79% water, while pure PLA absorbed about 0.28%. Similarly, Augustia et al. [76] found that High-Density Polyethylene/date palm fiber composites (5 wt%) had a water absorption of about 0.04%, compared to 0.017% for pure PLA. In general, biocomposites are reported to exhibit water absorption in the range of 0.7–2% after 24 h [77]. The higher absorption in the biocomposite is attributed to the hydrophilic nature of date palm fiber, which tends to attract and retain moisture [3].

The results of the thermal characterization, tensile testing, and water absorption studies are summarized in Table 3.

## 4. Relevance of Date Palm Fiber-Reinforced PLA for Sustainable Applications

The aim of this study is to emphasize both the sustainable processing route and the potential applications of PLA/date palm fiber biocomposites. The fabrication of PLA reinforced with date palm fibers is successfully achieved using extrusion and 3D Printing, demonstrating that this approach can be scaled up and commercialized for producing biodegradable composites. As highlighted by Khan et al. [78], date palm fiber is an eco-friendly alternative to fossil-based reinforcements and offers significant potential for sustainable applications.

Saudi Arabia alone generates over 200,000 tons of date palm biomass annually [78]. Most of this waste is either burned or landfilled, leading to serious environmental issues [79]. Utilizing this biomass in composite production not only provides an eco-friendly alternative to traditional plastics but also contributes to waste recycling and supports circular economy initiatives. Several studies have shown the suitability of date palm fibers for polymer reinforcement, particularly in packaging applications. For instance, Mousa et al. [80], developed PLA composites reinforced with date palm rachis microfibers for single-use packaging items such as cutlery.

PLA itself has gained wide attention in the packaging industry due to its compostability, good barrier properties against aroma and large molecules, and ability to preserve food freshness by preventing odor or flavor loss [52]. However, PLA has limitations, especially its slow degradation under natural conditions. While PLA decomposes in compost within weeks to months under high humidity and temperature, its environmental breakdown is limited [52,81]. Blending PLA with natural fibers helps overcome this drawback by enhancing its biodegradability [82,83,84]. Natural fibers also bring additional advantages: they are cost-effective, lightweight, renewable, and safer for both the environment and human health [77].

Although the tensile strength improvement observed in this study is modest (about 6.53%), several factors could explain this, including voids in the structure, uneven fiber distribution, and limited interfacial adhesion between fibers and the PLA matrix [85,86,87]. These limitations can be addressed through process optimization, including improved grinding, ball milling, filament extrusion, and 3D Printing parameters. In addition, fiber surface treatments can be applied to enhance fiber-matrix adhesion.

In addition to mechanical performance, this study also highlights other key aspects of date palm fiber-reinforced biocomposites, including sustainability, material processability, and thermal behavior. The incorporation of just 5 wt% date palm fiber, without any fiber treatment or major processing modifications, results in some improvement in tensile strength, showing that even low fiber loadings can be effective. Furthermore, the increase in tensile strength and reduction in elongation observed in this study are consistent with reports on natural fiber-reinforced PLA composites in the literature [56,88,89,90].

## 5. Conclusions

This study demonstrates the successful fabrication and characterization of a PLA/date palm fiber biocomposite filament containing 5 wt% date palm fiber, using extrusion and FDM-based 3D Printing. The incorporation of date palm fiber slightly modifies the thermal behavior of PLA, as indicated by DSC results. Both the biocomposite and pure PLA exhibit similar glass transition and melting temperatures. However, the biocomposite shows slightly higher thermal activity. Tensile testing reveals that the biocomposite improves yield and tensile strength by about 10.12% and 6.53%, respectively, compared to pure PLA, but exhibits reduced elongation at break, indicating increased brittleness. Fractographic analysis identifies inlayer fracture as the dominant failure mode and confirms that both PLA and the PLA/date palm fiber composite exhibit brittle failure. Water absorption is higher for the biocomposite (due to the hydrophilic nature of the date palm fiber) as compared to the pure PLA. The study also shows that reinforcing PLA with a small fraction (5 wt%) of date palm fibers, an abundant natural fiber resource, produces a cost-effective and environmentally friendly material. This biocomposite is particularly suitable for single-use plastic consumer products, such as packaging, disposable utensils, food containers, bags, etc., where biodegradability and sustainability are important.

Future studies will examine the effects of different date palm fiber percentages on the mechanical, thermal, and moisture resistance properties of the biocomposite. Further analysis is needed to evaluate compressive strength and impact resistance for a more comprehensive understanding of structural performance under various loads. The effects of extrusion and 3D Printing process parameters on print quality and structural integrity also require investigation. Additionally, future investigations can explore the impact of reinforcing polymers with a combination of date palm fiber and other natural or synthetic fillers to evaluate the resulting composite performance.

## Figures and Tables

**Table 1:** Extrusion process parameters for the filaments.

Parameters	Heater	Pure PLA	Biocomposite
Screw speed (rpm)		5	6.5
Fan cooling speed (%)		30	30
Feed zone (°C)	H4	170	170
Transition zone (°C)	H3	185	190
	H2	190	195
Metering zone (°C)	H1	180	180

**Table 2:** 3D Printing process parameters.

Parameters	Pure PLA	Biocomposite
Layer thickness (mm)	0.14	0.14
Print speed (mm/s)	70	63
Infill (%)	100	100
Platform temperature (°C)	55	65
Extrusion temperature (°C)	210	220

**Table 3:** Summary of Results.

Characterization	Properties	Pure PLA	Biocomposite
Thermal	Glass Transition Temperature (°C)	63.2	63
	Crystallization Temperature (°C)		124.1
	Melting Temperature (°C)	156.9	156.9
Mechanical	Yield Strength (MPa)	33.37 ± 0.92	36.75 ± 2.91
	Tensile Strength (MPa)	50.40 ± 0.62	53.69 ± 1.75
	Elongation at Break (%)	2.45 ± 0.20	1.93 ± 0.10
Physical	Water Absorption (%)	0.10 ± 0.014	0.58 ± 0.016

Note: The number following ± denotes the population standard deviation.

## Data Availability

The original contributions presented in this study are included in the article. Further inquiries can be directed to the corresponding author.

## Abbreviations

The following abbreviations are used in this manuscript:

PLA	Polylactic Acid
3D	Three-Dimensional
DSC	Differential Scanning Calorimetry
FDM	Fused Deposition Modeling
CAD	Computer-Aided Design
STL	Standard Tessellation Language
SEM	Scanning Electron Microscope
2D	Two-Dimensional

## References

[1] Olanrewaju O., Oladele I.O., Adelani S.O. (2025). Recent Advances in Natural Fiber Reinforced Metal/Ceramic/Polymer Composites: An Overview of the Structure-Property Relationship for Engineering Applications. Hybrid Adv..

[2] Ajithkumar S., Arulmurugan B., Rajeshkumar L., Puttegowda M., Gowda T.G.Y., Binoj J.B., Rangappa S.M., Siengchin S. (2025). 14—Prospects of Natural Fiber-Reinforced Polymer Composites in Engineering and Commercial Applications. Applications of Composite Materials in Engineering.

[3] Awad S., Hamouda T., Midani M., Katsou E., Fan M. (2023). Polylactic Acid (PLA) Reinforced with Date Palm Sheath Fiber Bio-Composites: Evaluation of Fiber Density, Geometry, and Content on the Physical and Mechanical Properties. J. Nat. Fibers.

[4] Trivedi A.K., Gupta M.K., Singh H. (2023). PLA Based Biocomposites for Sustainable Products: A Review. Adv. Ind. Eng. Polym. Res..

[5] Sarmin S.N., Jawaid M., Zaki S.A., Radzi A.M., Fouad H., Khiari R., Rahayu S., Amini M.H.M. (2023). Enhancing the Properties of Date Palm Fibre Reinforced Bio-Epoxy Composites with Chitosan—Synthesis, Mechanical Properties, and Dimensional Stability. J. King Saud Univ. Sci..

[6] Ghori W., Saba N., Jawaid M., Asim M. (2018). A Review on Date Palm (*Phoenix dactylifera*) Fibers and Its Polymer Composites. IOP Conf. Ser. Mater. Sci. Eng..

[7] AL-Oqla F.M., Midani M., Saba N., Alothman O.Y. (2020). Evaluation and Comparison of Date Palm Fibers with Other Common Natural Fibers. Date Palm Fiber Composites: Processing, Properties and Applications.

[8] AL-Oqla F.M., Sapuan S.M. (2018). Investigating the Inherent Characteristic/Performance Deterioration Interactions of Natural Fibers in Bio-Composites for Better Utilization of Resources. J. Polym. Environ..

[9] AL-Oqla F.M., Hayajneh M.T., Fares O. (2019). Investigating the Mechanical Thermal and Polymer Interfacial Characteristics of Jordanian Lignocellulosic Fibers to Demonstrate Their Capabilities for Sustainable Green Materials. J. Clean. Prod..

[10] Alaaeddin M.H., Sapuan S.M., Zuhri M.Y.M., Zainudin E.S., AL- Oqla F.M. (2019). Lightweight and Durable PVDF–SSPF Composites for Photovoltaics Backsheet Applications: Thermal, Optical and Technical Properties. Materials.

[11] Dhakal H.N., Khan S.H., Alnaser I.A., Karim M.R., Saifullah A., Zhang Z. (2024). Potential of Date Palm Fibers (DPFs) as a Sustainable Reinforcement for Bio-Composites and Its Property Enhancement for Key Applications: A Review. Macromol. Mater. Eng..

[12] Bamaga S.O. (2022). A Review on the Utilization of Date Palm Fibers as Inclusion in Concrete and Mortar. Fibers.

[13] Mlhem A., Abu-Jdayil B., Tong-Earn T., Iqbal M. (2022). Sustainable Heat Insulation Composites from Date Palm Fibre Reinforced Poly(β-Hydroxybutyrate). J. Build. Eng..

[14] Al Abdallah H., Abu-Jdayil B., Iqbal M.Z. (2022). The Effect of Alkaline Treatment on Poly(Lactic Acid)/Date Palm Wood Green Composites for Thermal Insulation. Polymers.

[15] Abu-Jdayil B., Barkhad M.S., Mourad A.-H.I., Iqbal M.Z. (2021). Date Palm Wood Waste-Based Composites for Green Thermal Insulation Boards. J. Build. Eng..

[16] Raza M., Al Abdallah H., Kozal M., Al Khaldi A., Ammar T., Abu-Jdayil B. (2023). Development and Characterization of Polystyrene–Date Palm Surface Fibers Composites for Sustainable Heat Insulation in Construction. J. Build. Eng..

[17] Kharrat F., Khlif M., Hilliou L., Haboussi M., Covas J.A., Nouri H., Bradai C. (2020). Minimally Processed Date Palm (*Phoenix dactylifera* L.) Leaves as Natural Fillers and Processing Aids in Poly(Lactic Acid) Composites Designed for the Extrusion Film Blowing of Thin Packages. Ind. Crops Prod..

[18] Maou S., Meghezzi A., Grohens Y., Meftah Y., Kervoelen A., Magueresse A. (2021). Effect of Various Chemical Modifications of Date Palm Fibers (DPFs) on the Thermo-Physical Properties of Polyvinyl Chloride (PVC)–High-Density Polyethylene (HDPE) Composites. Ind. Crops Prod..

[19] Pereira D.F., Branco A.C., Cláudio R., Marques A.C., Figueiredo-Pina C.G. (2023). Development of Composites of PLA Filled with Different Amounts of Rice Husk Fibers for Fused Deposition Modeling. J. Nat. Fibers.

[20] Kariz M., Sernek M., Obućina M., Kuzman M.K. (2018). Effect of Wood Content in FDM Filament on Properties of 3D Printed Parts. Mater. Today Commun..

[21] Xiao X., Chevali V.S., Song P., He D., Wang H. (2019). Polylactide/Hemp Hurd Biocomposites as Sustainable 3D Printing Feedstock. Compos. Sci. Technol..

[22] Kumar R., Srivastava P., Agrawal A.P., Kumar S., Kumar V. (2025). Development and Characterization of Polypropylene-Carbon Nanotubes (PP-CNT) Composites: An Overview Toward Hurdles and Achievements. Macromol. Symp..

[23] Srivastava P., Arumugam A.B., Agrawal A.P., Ntumba Z.N. (2025). Development of Hemp Fiber Reinforced PLA Composites for Sustainable 3D Printing: Mechanical and Microstructural Properties. J. Nat. Fibers.

[24] Mohanty A.K., Misra M., Hinrichsen G. (2000). Biofibres, Biodegradable Polymers and Biocomposites: An Overview. Macromol. Mater. Eng..

[25] Abdellah M.Y., Sadek M.G., Alharthi H., Abdel-Jaber G.T., Backar A.H. (2023). Characteristic Properties of Date-Palm Fibre/Sheep Wool Reinforced Polyester Composites. J. Bioresour. Bioprod..

[26] Ali M. (2023). Epoxy–Date Palm Fiber Composites: Study on Manufacturing and Properties. Int. J. Polym. Sci..

[27] Ali M., Al-Assaf A.H., Salah M. (2022). Date Palm Fiber-Reinforced Recycled Polymer Composites: Synthesis and Characterization. Adv. Polym. Technol..

[28] Aly R., Olalere O., Ryder A., Alyammahi M., Samad W.A. (2024). Mechanical Property Characterization of Virgin and Recycled PLA Blends in Single-Screw Filament Extrusion for 3D Printing. Polymers.

[29] 3devo Output Too Low or Thin. https://support.3devo.com/output-too-low-or-thin.

[30] 3devo Filament Thickness Deviation (Inconsistent Diameter). https://support.3devo.com/filament-thickness-deviation-inconsistent-diameter.

[31] 3devo FM Essentials: The Heaters. https://support.3devo.com/fm-essentials-the-heaters.

[32] (2021). Standard Test Method for Transition Temperatures and Enthalpies of Fusion and Crystallization of Polymers by Differential Scanning Calorimetry.

[33] Mulligan D., Gnaniah S., Sims G. (2003). Measurement Good Practice Guide No. 62—Thermal Analysis Techniques for Composites and Adhesives.

[34] Billah K.M.M., Lorenzana F.A.R., Martinez N.L., Chacon S., Wicker R.B., Espalin D. Thermal Analysis of Thermoplastic Materials Filled with Chopped Fiber for Large Area 3D Printing. Proceedings of the 30th Annual International Solid Freeform Fabrication Symposium.

[35] Karimi A., Rahmatabadi D., Baghani M. (2024). Various FDM Mechanisms Used in the Fabrication of Continuous-Fiber Reinforced Composites: A Review. Polymers.

[36] Khabia S., Jain K.K. (2020). Influence of Change in Layer Thickness on Mechanical Properties of Components 3D Printed on Zortrax M 200 FDM Printer with Z-ABS Filament Material & Accucraft I250+ FDM Printer with Low Cost ABS Filament Material. Mater. Today Proc..

[37] Zortrax M200—Award-Winning Desktop 3D Printer. https://zortrax.com/3d-printers/m200/.

[38] Mian S.H., Abouel Nasr E., Moiduddin K., Saleh M., Alkhalefah H. (2024). An Insight into the Characteristics of 3D Printed Polymer Materials for Orthoses Applications: Experimental Study. Polymers.

[39] Ismail R., Fitriyana D.F., Nugraha F.W., Bayuseno A.P., Ammarullah M.I. (2025). Investigation of the Influence of 3D Printing Parameters on the Properties of Interference Screws Made of PLA/PCL/HA Biocomposite Filaments. Mater. Technol..

[40] Wang S., Ma Y., Deng Z., Zhang S., Cai J. (2020). Effects of Fused Deposition Modeling Process Parameters on Tensile, Dynamic Mechanical Properties of 3D Printed Polylactic Acid Materials. Polym. Test..

[41] Alsoufi M.S., Alhazmi M.W., Suker D.K., Alghamdi T.A., Sabbagh R.A., Felemban M.A., Bazuhair F.K. (2019). Experimental Characterization of the Influence of Nozzle Temperature in FDM 3D Printed Pure PLA and Advanced PLA+. Am. J. Mech. Eng..

[42] Kumar M.S., Farooq M.U., Ross N.S., Yang C.-H., Kavimani V., Adediran A.A. (2023). Achieving Effective Interlayer Bonding of PLA Parts during the Material Extrusion Process with Enhanced Mechanical Properties. Sci. Rep..

[43] (2023). Standard Test Method for Tensile Properties of Plastics.

[44] Egan B.C., Brownell C.J., Murray M.M. (2016). Experimental Assessment of Performance Characteristics for Pitching Flexible Propulsors. J. Fluids Struct..

[45] Selvamani S.K., Rajan K., Samykano M., Kumar R.R., Kadirgama K., Mohan R.V. (2022). Investigation of Tensile Properties of PLA–Brass Composite Using FDM. Prog. Addit. Manuf..

[46] (2017). Tensile Properties of Rigid and Semi-Rigid Plastics (ASTM D638 and ISO 527).

[47] (2023). Tensile Properties ASTM D638 vs. ASTM D3039.

[48] Cooperstein I., Indukuri S.R.K.C., Bouketov A., Levy U., Magdassi S. (2020). 3D Printing of Micrometer-Sized Transparent Ceramics with On-Demand Optical-Gain Properties. Adv. Mater..

[49] Gong H., Crater C., Ordonez A., Ward C., Waller M., Ginn C. (2018). Material Properties and Shrinkage of 3D Printing Parts Using Ultrafuse Stainless Steel 316LX Filament. MATEC Web Conf..

[50] (2010). Standard Test Method for Water Absorption of Plastics.

[51] Kacem M.A., Guebailia M., Dezaki M.L., Abdi S., Sabba N., Zolfagharian A., Bodaghi M. (2025). Development and 3D Printing of PLA Bio-Composites Reinforced with Short Yucca Fibers and Enhanced Thermal and Dynamic Mechanical Performance. J. Mater. Res. Technol..

[52] Paul A., Sreedevi K., Sharma S.S., Anjana V.N. (2022). Polylactic Acid (PLA). Handbook of Biopolymers.

[53] Biosurfaces: A Materials Science and Engineering Perspective | Wiley. https://www.wiley.com/en-us/Biosurfaces%3A+A+Materials+Science+and+Engineering+Perspective-p-9781118299975.

[54] Cao M., Cui T., Yue Y., Li C., Guo X., Jia X., Wang B. (2022). Investigation of Carbon Fiber on the Tensile Property of FDM-Produced PLA Specimen. Polymers.

[55] Saleh M., Anwar S., AlFaify A.Y., Al-Ahmari A.M., Abd Elgawad A.E.E. (2024). Development of PLA/Recycled-Desized Carbon Fiber Composites for 3D Printing: Thermal, Mechanical, and Morphological Analyses. J. Mater. Res. Technol..

[56] Zadeh K.M., Inuwa I.M., Arjmandi R., Hassan A., Almaadeed M., Mohamad Z., Khanam P.N. (2017). Effects of Date Palm Leaf Fiber on the Thermal and Tensile Properties of Recycled Ternary Polyolefin Blend Composites. Fibers Polym..

[57] Araújo J.R., Waldman W.R., De Paoli M.A. (2008). Thermal Properties of High Density Polyethylene Composites with Natural Fibres: Coupling Agent Effect. Polym. Degrad. Stab..

[58] Mi Y., Chen X., Guo Q. (1997). Bamboo Fiber-Reinforced Polypropylene Composites: Crystallization and Interfacial Morphology. J. Appl. Polym. Sci..

[59] Andersson H., Örtegren J., Zhang R., Grauers M., Olin H. (2021). Variable Low-Density Polylactic Acid and Microsphere Composite Material for Additive Manufacturing. Addit. Manuf..

[60] 3devo PLA Colored Filament: Extrusion Walkthrough Using Masterbatches. https://support.3devo.com/colored-filament.

[61] Fouly A., Alnaser I.A., Assaifan A.K., Abdo H.S. (2022). Evaluating the Performance of 3D-Printed PLA Reinforced with Date Pit Particles for Its Suitability as an Acetabular Liner in Artificial Hip Joints. Polymers.

[62] Neoh K.W., Tshai K.Y., Khiew P.S., Chia C.H. (2012). Micro Palm and Kenaf Fibers Reinforced PLA Composite: Effect of Volume Fraction on Tensile Strength. Appl. Mech. Mater..

[63] Ghanmi I., Slimani F., Ghanmi S., Guedri M. (2024). Development and Characterization of a PLA Biocomposite Reinforced with Date Palm Fibers. Eng. Technol. Appl. Sci. Res..

[64] Ebrahim A., Abdellah M.Y., Gomaa A.M.A., Kourmpetis M., Youssef H.A.H., Abdel-Jaber G.T. (2025). Enhancing Polymer Composites with Date Palm Residues for Sustainable Innovation: A Review. Int. J. Mater. Res..

[65] Khosravani M.R., Berto F., Ayatollahi M.R., Reinicke T. (2022). Characterization of 3D-Printed PLA Parts with Different Raster Orientations and Printing Speeds. Sci. Rep..

[66] Yao T., Ye J., Deng Z., Zhang K., Ma Y., Ouyang H. (2020). Tensile Failure Strength and Separation Angle of FDM 3D Printing PLA Material: Experimental and Theoretical Analyses. Compos. Part B Eng..

[67] Gao H., Qiang T. (2017). Fracture Surface Morphology and Impact Strength of Cellulose/PLA Composites. Materials.

[68] Cabrera F.L., Arellano M.T., Franco-Urquiza E.A. (2025). PLA/Jute Fabric Laminate Composites: Influence of Weight Percentage on Mechanical Properties and Fracture Behavior. Discov. Mech. Eng..

[69] Kaiser M., Anuar H., Razak S. (2013). Ductile–Brittle Transition Temperature of Polylactic Acid-Based Biocomposite. J. Thermoplast. Compos. Mater..

[70] Azad M.M., Shah A.u.R., Prabhakar M.N., Kim H.S. (2024). Deep Learning-Based Microscopic Damage Assessment of Fiber-Reinforced Polymer Composites. Materials.

[71] Mukherjee T., Kao N. (2011). PLA Based Biopolymer Reinforced with Natural Fibre: A Review. J. Polym. Environ..

[72] Banjo A.D., Agrawal V., Auad M.L., Celestine A.-D.N. (2022). Moisture-Induced Changes in the Mechanical Behavior of 3D Printed Polymers. Compos. Part C Open Access.

[73] Ecker J.V., Haider A., Burzic I., Huber A., Eder G., Hild S. (2019). Mechanical Properties and Water Absorption Behaviour of PLA and PLA/Wood Composites Prepared by 3D Printing and Injection Moulding. Rapid Prototyp. J..

[74] Schwarz D., Pagáč M., Petruš J., Polzer S. (2022). Effect of Water-Induced and Physical Aging on Mechanical Properties of 3D Printed Elastomeric Polyurethane. Polymers.

[75] Hall D.C., Palmer P., Ji H.-F., Ehrlich G.D., Król J.E. (2021). Bacterial Biofilm Growth on 3D-Printed Materials. Front. Microbiol..

[76] Augustia V.A.S., Chafidz A., Setyaningsih L., Rizal M., Kaavessina M., Zahrani S.M.A. (2018). Effect of Date Palm Fiber Loadings on the Mechanical Properties of High Density Polyethylene/Date Palm Fiber Composites. Key Eng. Mater..

[77] Mohammed M., Jawad A.J.M., Mohammed A.M., Oleiwi J.K., Adam T., Osman A.F., Dahham O.S., Betar B.O., Gopinath S.C.B., Jaafar M. (2023). Challenges and Advancement in Water Absorption of Natural Fiber-Reinforced Polymer Composites. Polym. Test..

[78] Khan S.H., Dhakal H.N., Saifullah A., Zhang Z. (2025). Improved Mechanical and Thermal Properties of Date Palm Microfiber-Reinforced PCL Biocomposites for Rigid Packaging. Molecules.

[79] Belgacem C., Serra-Parareda F., Tarrés Q., Mutjé P., Delgado-Aguilar M., Boufi S. (2021). The Integral Utilization of Date Palm Waste to Produce Plastic Composites. Polymers.

[80] Mousa N., Galiwango E., Haris S., Al-Marzouqi A.H., Abu-Jdayil B., Caires Y.L. (2022). A New Green Composite Based on Plasticized Polylactic Acid Mixed with Date Palm Waste for Single-Use Plastics Applications. Polymers.

[81] Satti S.M., Shah A.A., Marsh T.L., Auras R. (2018). Biodegradation of Poly(Lactic Acid) in Soil Microcosms at Ambient Temperature: Evaluation of Natural Attenuation, Bio-Augmentation and Bio-Stimulation. J. Polym. Environ..

[82] Kalita N.K., Damare N.A., Hazarika D., Bhagabati P., Kalamdhad A., Katiyar V. (2021). Biodegradation and Characterization Study of Compostable PLA Bioplastic Containing Algae Biomass as Potential Degradation Accelerator. Environ. Chall..

[83] Wan Ishak W.H., Rosli N.A., Ahmad I. (2020). Influence of Amorphous Cellulose on Mechanical, Thermal, and Hydrolytic Degradation of Poly(Lactic Acid) Biocomposites. Sci. Rep..

[84] Yu M., Zheng Y., Tian J. (2020). Study on the Biodegradability of Modified Starch/Polylactic Acid (PLA) Composite Materials. RSC Adv..

[85] Moorthy J.S.N., Chandran M.S. (2025). A Comprehensive Review on the Influence of Surface Treatment and 3D Printing of Natural Fiber Composites. Compos. Interfaces.

[86] Mundhe A., Kandasubramanian B. (2024). Advancements in Natural Fiber Composites: Innovative Chemical Surface Treatments, Characterizaton Techniques, Environmental Sustainability, and Wide-Ranging Applications. Hybrid Adv..

[87] Ilyas R.A., Zuhri M.Y.M., Aisyah H.A., Asyraf M.R.M., Hassan S.A., Zainudin E.S., Sapuan S.M., Sharma S., Bangar S.P., Jumaidin R. (2022). Natural Fiber-Reinforced Polylactic Acid, Polylactic Acid Blends and Their Composites for Advanced Applications. Polymers.

[88] Kunanopparat T., Menut P., Morel M.-H., Guilbert S. (2008). Reinforcement of Plasticized Wheat Gluten with Natural Fibers: From Mechanical Improvement to Deplasticizing Effect. Compos. Part A Appl. Sci. Manuf..

[89] Ma X., Yu J., Kennedy J.F. (2005). Studies on the Properties of Natural Fibers-Reinforced Thermoplastic Starch Composites. Carbohydr. Polym..

[90] Nur H.P., Hossain M.A., Sultana S., Mollah M.M. (2010). Preparation of Polymer Composites Using Natural Fiber and Their Physico-Mechanical Properties. Bangladesh J. Sci. Ind. Res..

